# Comparative proteomics: assessment of biological variability and dataset comparability

**DOI:** 10.1186/s12859-015-0561-9

**Published:** 2015-04-17

**Authors:** Sa Rang Kim, Tuong Vi Nguyen, Na Ri Seo, Seunghup Jung, Hyun Joo An, David A Mills, Jae Han Kim

**Affiliations:** 1 Department of Food and Nutrition, Chungnam National University, Daejeon, 305-764 South Korea; 2 Graduate School of Analytical Science and Technology, Chungnam National University, Daejeon, 305-764 South Korea; 3 Robert Mondavi Institute for Wine and Food Science, Department of Food Science, University of California, Davis, CA 95616 USA

**Keywords:** Comparative proteomics, Whole cell proteome, Internal standard, *Loctococcus lactis*

## Abstract

**Background:**

Comparative proteomics in bacteria are often hampered by the differential nature of dataset quality and/or inherent biological deviations. Although common practice compensates by reproducing and normalizing datasets from a single sample, the degree of certainty is limited in comparison of multiple dataset. To surmount these limitations, we introduce a two-step assessment criterion using: (1) the relative number of total spectra (*R*_*TS*_) to determine if two LC-MS/MS datasets are comparable and (2) nine glycolytic enzymes as internal standards for a more accurate calculation of relative amount of proteins. *Lactococcus lactis* HR279 and JHK24 strains expressing high or low levels (respectively) of green fluorescent protein (GFP) were used for the model system. GFP abundance was determined by spectral counting and direct fluorescence measurements. Statistical analysis determined relative GFP quantity obtained from our approach matched values obtained from fluorescence measurements.

**Results:**

*L. lactis* HR279 and JHK24 demonstrates two datasets with an *R*_*TS*_ value less than 1.4 accurately reflects relative differences in GFP levels between high and low expression strains. Without prior consideration of *R*_*TS*_ and the use of internal standards, the relative increase in GFP calculated by spectral counting method was 3.92 ± 1.14 fold, which is not correlated with the value determined by the direct fluorescence measurement (2.86 ± 0.42 fold) with the *p* = 0.024. In contrast, 2.88 ± 0.92 fold was obtained by our approach showing a statistically insignificant difference (*p* = 0.95).

**Conclusions:**

Our two-step assessment demonstrates a useful approach to: (1) validate the comparability of two mass spectrometric datasets and (2) accurately calculate the relative amount of proteins between proteomic datasets.

**Electronic supplementary material:**

The online version of this article (doi:10.1186/s12859-015-0561-9) contains supplementary material, which is available to authorized users.

## Background

Most research in biology relies on comparative observations of two or more conditions in a quantitative or descriptive manner [1]. Quantitative measurements (isotope labeling, label free methods, etc.) in comparative proteomics have been explored [2,3], but it is important to determine whether two datasets derived from different experimental conditions can be compared. Comparability (qualitative similarity of datasets) should be assessed prior to the quantitative comparison of LC-MS/MS datasets.

Data normalization across samples from different biological conditions is another critical point of comparative proteomics. Individual datasets from LC-MS/MS can be obtained with careful sample preparation and mass spectrometry application (injection volume, injection concentration, reproducibility, etc.) to minimize deviations between samples. However, sample deviation is often fundamental and originates from different biological conditions and cannot be assessed by the extant reproducibility of any one sample or dataset normalization.

To approach such problems in comparative proteomics, we hypothesized that proteins expressed consistently across various cellular conditions can be used as internal standards for quantification as well as a dataset comparability indicator. Genes in the glycolytic pathway are widely used as internal standards to normalize DNA microarrays and quantitative PCR studies and were ideal for our purpose. This study selected constitutively expressed proteins from *Lactococcus lactis*’ glycolytic pathway as internal standards for comparative proteomic analyses [4-10].

We employed a simple, applicable, and accurate spectral counting method demonstrated by numerous researchers to quantify proteins [5,11-16]. This spectral counting method is particularly useful with protein mixtures and whole cell proteomic analyses [1]. The relative amount of a protein between two samples was estimated by comparing two normalized spectral abundance factors (NSAF).

We assessed the approach’s reliability by comparing whole cell proteomes and relative amount of green fluorescent protein (GFP) from two strains expressing GFP at low or high levels, *L. lactis* HR279 and JHK24 respectively [17-19]. The plasmid pHR086 present in HR279 is an *Escherichia coli*- *L. lactis* shuttle vector containing a nisin-inducible GFP expression cassette and pJH24 present in JHK24 is the high copy variant of pHR086. A previous comparative protein expression study demonstrated these high and low copy vectors showed strong correlation between GFP fluorescence intensity and GFP amount per cell [18].

In this study, relative increases in GFP expression among whole *L. lactis* cell proteomes was calculated using the number of GFP’s MS/MS spectra and the comparison to nine internal standards. Relative increase determined by spectral counting was then compared to values obtained from GFP fluorescence emission. LC-MS/MS dataset reproducibility from one sample and dataset comparability between two samples was evaluated using internal standards. We define relative number of total spectra (*R*_*TS*_) as a presumptive parameter to evaluate mass spectrometric (MS) dataset’s comparability. In addition, our statistical analysis illustrates the importance of assessing a dataset’s comparability before calculating the relative protein quantities between two proteomic datasets.

## Results and discussion

### Strategy for the comparability assessment using internal standards

We define ‘comparability’ as the determination of whether two datasets have similar quality in order to correctly reflect proteomic changes occurring between two experimental conditions. Data reproducibility is key in determining comparability between single sample analytical replicates. Ideally, the change (*ave_SRA*[*rep, k*]) and standard deviation (*SD_SRA*[*rep, k*]) from averaged relative amounts of each protein from whole proteome biological replicates is 1.0 and 0, respectively. In this case, standard deviation reflects reproducibility between replicates.

The comparability assessment, however, becomes problematic when two samples from different experimental conditions are compared. Because datasets from two independent experimental conditions inherently exhibit a difference in each protein’s amount; the standard deviation of the relative amount of total *protein (SD_SRA[comp*, k]) should not be directly used as a dataset comparability assessment parameter. Instead, a subset of consistently expressed proteins should serve as internal standards for this quality assessment. Consistent expression levels in two different experimental conditions infer that internal standards used are “pseudo-replicates” shared by the two samples. Therefore, the standard deviation of the relative amount of internal standards (*SD_SRA*[*comp, INj*]) should be used in comparability assessments between two samples from different experimental conditions.

We also define ‘relative number of total spectra’ (*R*_*TS*_) as an additional parameter to assess dataset comparability prior to calculating each protein’s relative amount. Linear correlation between *R*_*TS*_ and the standard deviation of internal standards (*SD_SRA*[*comp, INj*] and *SD_SRA*[*rep, INj*]) helped determine *R*_*TS*_ threshold value for the comparability assessment that permits a viable comparison.

### Results of LC-MS/MS data and identification of proteins

**Figure 1:**
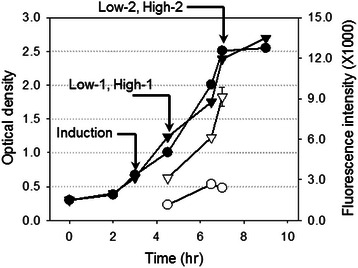
Cell growth curve of *L. lactis* HR279 (triangle) and JHK24 (circle). The fluorescence from the HR279 and JHK24 are depicted as open triangles and open circles. The arrows indicated the induction of GFP expression by adding a nisin and the sample collection points.

Our experimental system employed four different conditions (Figure 1), *L. lactis* cells containing either a high or low copy plasmid expressing GFP, sampled at exponential phase (High-1, Low-1, respectively) and early stationary phase (High-2, Low-2, respectively). Three biological replicates of each sample were prepared in the separate sets of experiments. The replicates referred in this work are biological replicates, not analytical or technical replicates of a single biological sample. The samples and the total number of the MS/MS spectra used to identify the proteins (*SpC*_*total*_) are summarized in Table 1. The biological replicates from the early exponential phase samples, High-1 and Low-1, exhibited a *SpC*_*total*_ ranging from 2406 to 4514 resulting in a large value of *R*_*TS*_ (1.20 ~ 1.79). In contrast, the *SpC*_*total*_ of the early stationary phase samples, High-2 and Low-2, had more uniform numbers between 3810 and 4492 and, consequently, a low *R*_*TS*_ value close to 1.0.

**Table 1:** **The summary of the LC-MS/MS results**

**Samples**	**Growth phase**	***SpC***_***total***_^***a***^			***R***_***TS***_^***b***^			**Number of identified proteins** ^***c***^							**Total** ^***d***^
		**A**	**B**	**C**	**A/B**	**A/C**	**B/C**	**A∩B∩C**	**A∩B**	**A∩C**	**B∩C**	**A**	**B**	**C**	
***L. lactis*** **JHK24**															
**High-1**	exp^*e*^	2406	2878	4150	1.20	1.72	1.44	221	7	11	20	4	4	17	284
**High-2**	stat ^*f*^	4492	4362	4347	1.03	1.03	1.00	262	18	9	6	2	4	2	303
***L. lactis*** **HR279**															
**Low-1**	exp	3226	2522	4514	1.28	1.40	1.79	233	7	26	21	1	3	17	308
**Low-2**	stat	3810	4339	4259	1.14	1.12	1.02	242	6	6	43	4	9	5	315

^*a*^ *SpC*_*total*_ is a total number of MS/MS spectra used to identify proteins in sample. False discovery rates (FDR) of peptides were calculated by searching the MS/MS spectra against the forward and the reversed entry database independently. The searching was performed with the 1% of FDR level.

^*b*^ *R*_*TS*_ is the relative number of total spectra.

^*c*^The A, B and C represent the replicates of each sample., A∩B∩C and A∩B represent the number of proteins appeared in all triplicates and two (A and B) out of three replicates, respectively.

^*d*^Total indicated the number of proteins identified from biological replicates. The guideline of protein identification was described in Result section. The number of unique peptide, X! Tandem value and the number presence among replicates were used as a parameter to decide the presence of proteins.

^e,*f*^Bacterial cell was taken at the early exponential and early stationary phase of cell growth stage, respectively.

As shown in Table 1, approximately 300 proteins were determined from each sample of three biological replicates. Between 76% to 86% of proteins were present in all biological replicates, and more than 90% of proteins appeared in at least two of the three biological replicates. Replicates with a small *R*_*TS*_ value (for example, the sample High-2) showed only 8 proteins uniquely detected among individual replicates. However, the replicates of Low-1, which showed a large *R*_*TS*_, exhibited 25 proteins that were uniquely present in only one of the three biological replicates.

### Biological variability

**Figure 2:**
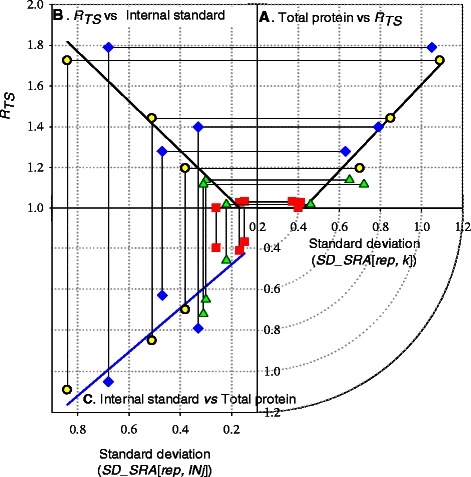
Correlation between *R*_*TS*_ and variability of biological replicates. Three way correlations on 3D space was projected on each xyz-plane describing the linear correlation between **(A)** *R*_*TS*_ and standard deviation of total proteins (*SD_SRA*[*rep, k*]), **(B)** *R*_*TS*_ and standard deviation of internal standards (*SD_SRA*[*rep, INj*]), and **(C)** standard deviation of total protein (*SD_SRA*[*rep, k*]) and internal standards (*SD_SRA*[*rep, INj*]). The X-axis of graph **(A)** and Y-axis of graph **(C)** shard the same value and range of *SD_SRA*[*rep, k*]. The circles, squares, diamonds, and triangles represent comparisons between biological replicates of samples High-1, High-2, Low-1 and Low-2 in Table 2, respectively.

*SD_SRA*[*rep, k*] variation has been used for the indication of the quantitative reproducibility between sample replicates. Biological replicates of High-2 exhibited strong quantitative consistency with a 0.37-0.41-fold standard deviation. However, High-1 replicates exhibited a wider range of standard deviations (0.7-1.09 fold; Additional file 1: Table S1; Figure 2 - Correlation between *R*_*TS*_ and variability of biological replicates.). Under two replicates’ ideal reproducibility, *SRA*[*rep, k*] would exhibit a value of zero (i.e. a change of 1.0-fold) or commonly a single value. However, when *SRA*[*rep, k*] exhibits a normal distribution (Additional file 2: Figure S1); the reproducibility can be measured by *SD_SRA*[*rep, k*] of the *SRA*[*rep, k*] distribution.

Once MS analytical reproducibility is guaranteed, dataset quality variation occurs mainly due to biological variability between samples. Dataset normalization tools such as NSAF or technical replicates cannot adjust for the variation between samples; resulting in inaccurate quantitation unless dataset quality is the same. Injecting the same amount of protein to MS can minimize variability in dataset quality but does not necessarily reflect the same initial biological specimen amount. For example, cell protein recovery varies depending on the morphology (cell wall rigidity, exopolysaccharide production) and total amount of protein per cell. Cell lysate’s protein concentrations obtained from growth on different substrates or taken at different growth stages differed up to 3.9-fold even though the same number of cells (as determined by optical density) were initially used (Additional file 1: Table S2). Thus, normalization using protein amounts injected into the MS analysis cannot adjust variation from samples’ different biological conditions.

### Internal standards

The nine glycolytic proteins chosen as internal standards exhibited smaller quantitative variations than the total protein pool (Additional file 1: Table S1). *SD_SRA*[*rep, INj*] between replicate of the High-2 samples were 0.15- 0.27 fold and showed good reproducibility. High-1’s biological replicates still showed a higher value of *SD_SRA*[*rep, INj*] (0.34- to 0.84 fold). Figure 2A presents *SD_SRA*[*rep, INj*] linearly correlated to total proteins (*SD_SRA*[*rep, k*]), exhibiting a correlation coefficient of 0.84.

Glycolytic enzymes were chosen in this particular study since they are constitutively expressed throughout the cell growth. Alternative sets of internal standards could be used in different experimental conditions or biological systems. For example, the enzymes involved in the Calvin cycle can be used as internal standards for the plant cell. Comparative proteomics between wild type (W) and the mutant (KE) strains of *Oryza sativa* subsp*. japonica* showed that the enzymes involved in the photosynthesis were constitutively expressed (Additional file 1: Table S3). In the individual comparisons between the replicates of W and KE strains, the *SD_SRA*[*comp, INj*] of selected enzymes were between 0.19- to 0.43-fold suggesting constitutive expression and, consequently, potential use as internal standards (Additional file 1: Table S4)

A common practice in mRNA expression studies is to normalize the expression levels to one or more internal standard(s) [4,6,7,9,10]. This controls for sample variation due to differences in RNA preparation. We hypothesized that the same approach would work in comparative proteomics and used several constitutively expressed proteins from the glycolytic pathway of *L. lactis* as internal standards. Alternative sets of internal standards could be used in different experimental conditions or biological systems. Indeed, the narrow range of standard deviations in the relative amounts of nine glycolytic enzymes at different growth stages (*SD_SRA*[*comp*, *INj*]) suggests that the expression level of nine enzymes in two different strains and two independent growth stages of *L. lactis* were maintained at a constant level (Table 2) and did not correlated to the R_TS_ values (Additional file 2:Figure 2S).

**Table 2:** **Relative amount of GFP between high and low expression system at different stage of cell growth**

**Samples**	**Comparison** ^**a**^	***R***_***TS***_	**Internal standard** ^**b**^	**Relative amount of GFP**	
				**LC-MS/MS** ^**c**^	**FL** ^**d**^
High-1 *vs* Low-1	1A/3A	1.34	−1.43 ± 0.44	2.49 ± 0.57	2.86 ± 0.42
	1B/3A	1.12	−1.03 ± 0.32	2.66 ± 0.71	
	1C/3A	1.29	−1.07 ± 0.38	2.51 ± 0.75	
	1A/3B	1.05	−1.23 ± 0.66	3.88 ± 1.69	
	1B/3B	1.14	1.23 ± 0.53	2.12 ± 0.66	
	1C/3B	1.65	1.22 ± 0.77	2.06 ± 0.94	
	1A/3C	1.88	−1.44 ± 0.87	7.09 ± 3.17	
	1B/3C	1.57	−1.01 ± 0.51	3.89 ± 1.23	
	1C/3C	1.09	1.35 ± 0.44	3.48 ± 0.59	
High-2 *vs* Low-2	2A/4A	1.18	−1.09 ± 0.27	3.27 ± 0.70	4.00 ± 0.62
	2B/4A	1.14	−1.12 ± 0.37	3.66 ± 1.08	
	2C/4A	1.14	−1.14 ± 0.34	4.00 ± 1.09	
	2A/4B	1.04	−1.04 ± 0.19	3.87 ± 0.61	
	2B/4B	1.01	−1.06 ± 0.20	4.28 ± 0.76	
	2C/4B	1.00	−1.09 ± 0.27	4.73 ± 1.10	
	2A/4C	1.05	−1.06 ± 0.23	3.80 ± 0.79	
	2B/4C	1.02	−1.08 ± 0.30	4.24 ± 1.06	
	2C/4C	1.02	−1.05 ± 0.19	4.58 ± 0.89	

^a^Number indicates the group of sample and A, B, and C indicates the biological replicates as described in Table 1.

^b^Internal standard is the average relative amount of internal standards between two biological replicates.

^c^The relative amount of GFP between two biological replicates of samples calculated by the spectral counting method with the use of internal standards.

^d^Fluorescence was measured in triplicate from separate biological replicates.

### Comparability assessment

The comparability assessment of two samples obtained from different biological conditions starts with comparing the two constitutively expressed internal standards (glycolytic enzymes) subsets. Threshold value obtained from the analysis of replicates (0.46-fold) is applied to assess the comparability between two independent sample sets.

We used standard deviations from biological replicates to design an acceptable range for our comparability assessment. The minimum *SD_SRA*[*rep, k*] obtained was 0.38-fold. This experimental observation led us to a determined threshold for the comparability assessment: a standard deviation difference of 0.76-fold (twice the experimentally determined minimum value). Standard deviations of 0.38- and 0.76-fold are equivalent to linear correlation coefficients of 0.98 and 0.95, respectively, when each protein’s NSAF was plotted on a log-log plot (Additional file 2: Figure S3). Consequently, the *SD_SRA*[*rep, INj*] was 0.46-fold; this became our threshold for comparability assessments from standard deviation correlations between internal standards and total protein (Figure 2).

### *R*_*TS*_- LC data quality

While internal standards are capable of adjusting biological variability between two independent samples, sample comparability should be assessed prior to calculations. *R*_*TS*_ is a presumptive parameter for quality assessment correlating with *SD_SRA*[*comp, INj*].

Biological replicates from exponential phase samples (High-1, Low-1) exhibited *SpC*_*total*_ of 2406–4514 resulting in larger *R*_*TS*_ values (1.20-1.79). In contrast, early stationary phase samples (high-2, low-2) *SpC*_*total*_ had more uniform numbers between 3810 and 4492 and, consequently, lower *R*_*TS*_ values closer to 1.0.

**Figure 3:**
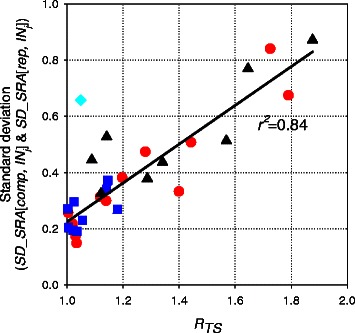
Correlation of *R*_*TS*_ and the standard deviation of a relative amount of internal standards. Correlation of *R*_*TS*_ and the standard deviation of a relative amount of internal standards between replicates (*SD_SRA*[*rep, INj*]) and between two samples (*SD_SRA*[*comp, INj*]). The standard deviations obtained from the comparison of High-1vs Low-1, High-2 *vs.* Low-2 and replicates in each group are shown as squares, triangles and circles, respectively. The diamonds depict the outliers from the comparisons between the sample High-1 and Low-1.

*R*_*TS*_ positively correlated with *SD_SRA*[*rep, k*]: *r* ^*2*^ = 0.89 and *SD_SRA*[*rep, INj*]: *r* ^*2*^ = 0.86 (Figure 2B & 2C). Increasing the value of *R*_*TS*_ increased the *SD_SRA*[*rep, k*] value; suggesting poor dataset comparability at higher *R*_*TS*_ values. The calculated threshold for the comparability assessment was *R*_*TS*_ of 1.35 using linear correlation where *SD_SRA*[*rep, k*] and *SD_SRA*[*rep, INj*] were 0.76- and 0.46-fold, respectively. *R*_*TS*_ exhibited linear correlations with the standard deviations of the internal standard proteins from replicates within one sample (*SD_SRA*[*rep, INj*]) and between replicates of two different samples (*SD_SRA*[*comp, INj*]) (Figure 3). The correlation (*r* ^*2*^ = 0.84) allowed us to calculate an *R*_*TS*_ value of 1.34 using a standard deviation of 0.46-fold; showing good agreement with values obtained from biological replicates.

*R*_*TS*_ values close to 1.0 exhibited similar ion chromatograms and protein identification results (Table 1). In addition, *R*_*TS*_ values correlated to the quantitative reproducibility of each protein in the replicates. *SD_SRA*[*rep*, *k*] and *SD_SRA*[*rep*, *INj*] between replicates linearly correlates to the *R*_*TS*_ value (Figure 2). These observations support the notion that the *R*_*TS*_ value is able to represent the quality of two MS datasets. The *R*_*TS*_ value was introduced to assess the dataset comparability prior to the calculation because it contains information on the sample’s absolute quantity of peptide identification information which *SD_SRA*[*comp, INj*] does not.

The correlation between *R*_*TS*_ and the quantitative reproducibility has been evaluated using the public database. Six replicates of *Escherichia coli* whole cell proteomic datasets were retrieved from the proteomeXchange website (http://www.proteomexchange.org/) [20]. The *Ave_SRA*[*rep*, *k*] between six replicates were within ±1.77 folds, however, the *SD_SRA*[*rep*, *k*] exhibited wide ranges from 0.5-fold to 3.0-fold depending on the dataset quality. From the *SD_SRA*[*rep*, *INj*], we were able to validate *R*_*TS*_ as a the quality assessment parameter (Additional file 2: Figure S4)

Quality levels between MS datasets of two samples plays a critical role in relative protein quantitation. When a good quality sample (many proteins identified, large number of SpC_total_), is compared with a poor quality sample (few identified proteins, small SpC_total_), calculating the relative protein amount can be more inaccurate than comparing two poor quality samples as demonstrated in our data. SpC_total_ from biological replicate A of High-1 was 2406 (1A; poor quality), biological replicate B and C of Low-1 sample had of 2878 (3B; poor quality) and 4150 (3C; good quality), respectively. GFP’s relative amount calculated using 1A/3B was 3.88 ± 1.69 fold; closer to the actual value measured by fluorescence (2.86 ± 0.42 fold) than the comparison between 1A/3C (7.09 ± 3.17 fold).

### GFP expression

**Figure 4:**
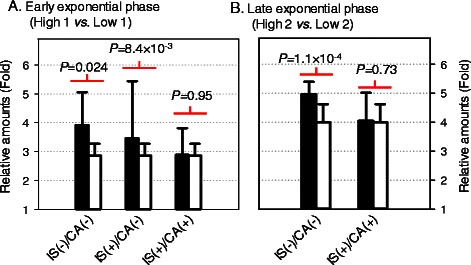
The impact of comparability assessment and the use of internal standards. The relative increase of GFP expression calculated by the spectral counting method (black bar) was compared to that obtained by the external measurement using fluorescent (white bar). IS and CA represented the use of internal standards and comparability assessment, and the sign of plus (+) and minus (−) indicates the “the use” and “without the use” of method, respectively. For example, IS(+)/CA(+) meant the relative amount of GFP calculated with the use of internal standard and comparability assessment of replicates. The *p*-values were obtained by the Student *t*-test (see the Experimental procedures). **(A)** and **(B)** is the comparison of sample obtained at early and late exponential phase, respectively.

The bioinformatical analysis about the comparability and quality assessment was validated by the biological experiment using GFP expression. Fluorescence determined the relative amount of GFP between High-1 and Low-1 to be 2.86 ± 0.42 fold (Figure 4A). GFP expression increase between High and Low samples was 3.92-fold with a standard deviation of ±1.14 fold when the comparability assessment criteria was not used (Figure 4A; IS(−)/CA(−)). This is 140% higher and contains a larger standard deviation than values obtained from fluorescence measurements. A student’s *T* test calculated a *p*-value of 2.4 × 10^−2^ (IS(−)/CA(−)) and 8.4 × 10^−3^ (IS(+)/CA(−)) were obtained when internal standard and comparability assessment were not employed (Figure 4A).

Comparisons between replicates showed increases ranging from 2.06 ± 0.94 to 7.09 ± 3.14 fold (average 3.46 ± 1.97 fold increase) when internal standards were applied to protein quantification (Table 2 & Figure 4A). Although the calculated accuracy value improved, the standard deviation was still large (RSD of 57%). However, our comparability criteria eliminates MS dataset outliers (7.09 ± 3.17 or 2.06 ± 0.94) resulting in a smaller range for the relative amount of GFP (2.12 ± 0.66 to 3.48 ± 0.59 fold). The pooled value of relative GFP increase between comparable datasets (IS(+)/CA(+)) was 2.88 ± 0.92 fold; showing good correlation with the value obtained from external fluorescence measurements (Figure 4A). Student’s *t*-test showed a *p*-value of 0.95 between values obtained from external measurement and our mass spectrometric approach.

GFP’s relative increase amount between High-2 and Low-2 were more uniform (Table 2). Comparisons between replicates had *SD_SRA*[*comp, INj*] values lower than 0.46-fold, suggesting good comparability. GFP expression increase between each biological replicates (IS(+)) were in the range of 3.27 ± 0.70 to 4.73 ± 1.10 fold; numbers similar to the 4.00 ± 0.62 fold increase obtained from the external fluorescence measurements (Table 2). Pooled relative increase of GFP expression from MS dataset was 4.05 ± 0.97 fold (Figure 4B; IS(+)/CA(+)), correlating to values observed via fluorescence with a *p*-value of 0.73. However, the relative GFP amount was 4.69 ± 0.44 fold and the *p*-value was 1.1 × 10^−4^ without the use of internal standards (Figure 4B; IS(−)/CA(−)).

## Conclusions

We have developed and discussed a novel method to accurately calculate a protein’s relative quantity in two whole cell proteomes. We determined that a preliminary proteomic dataset screen is necessary before an accurate relative protein abundance comparison can be made. In particular, we showed that assessing the relative number of total spectra (*R*_*TS*_) between two datasets can forecast the quality of the ensuing quantitative comparison. Once two datasets were deemed comparable, we used nine glycolytic enzymes as internal standards to calculate protein relative abundance in the two proteomes. We used GFP as a model protein to demonstrate GFP’s relative abundance. Any two proteomic datasets with a *R*_*TS*_ value less than 1.4 produced a value in close agreement with direct GFP fluorescence measurements (*p*-value of 0.73 and 0.95). While methods developed here employ spectral counting, their application is not limited to the label-free approaches.

## Methods

### Cell culture

*L. lactis* HR279 and *L. lactis* JHK24 were cultivated in M17 media (BD, Franklin Lakes, NJ) containing 3% (w/v) glucose (M17-G) supplemented with 5 μg/ml erythromycin (Sigma, St. Louis, MO). Fermentations were initiated by inoculating 5 ml seed culture and incubated at 30°C without shaking. M17-G media’s volume and initial pH were 300 ml and 6.5, respectively. pH was not controlled during the fermentations. The optical density (OD) was measured by a Beckman DU 7400 spectrophotometer (Beckman, Fullerton, CA) at 600 nm. Green fluorescent protein (GFP) expression was induced by adding nisin to a final concentration of 25 ng/ml when cell OD reached 0.7. GFP expression was monitored by measuring cell fluorescence. Cell pellets were washed three times in phosphate-buffered saline (PBS) and normalized to an OD_600nm_ of 0.1 before analysis. Fluorescence from 100 μl of normalized cells was measured in an ABI770 real time thermo cycler using excitation and emission wavelengths of 488 nm and 520 nm, respectively [19].

### Sample preparation and protein digestion

Fifty milliliters of *L. lactis* in media was removed at early and late exponential phase of cell growth as indicated in Figure 1. Early and late exponential phase samples from *L. lactis* HR279 were designated as Low-1 and Low-2 and samples from *L. lactis* JHK24 were High-1 and High-2, respectively. Initial cell mass amounts were normalized to an OD_600nm_ of 1.0 by dilution or concentration to a final volume of 25 ml. Cells underwent centrifugation, washing (three times with PBS), and resuspension (1 ml of lysis buffer containing 100 mM Tris and 8.0 M urea, pH 9.0). Cell disruption used 300 μg silica beads (Sigma-Aldrich, ST. Louis, MO) and a bead beater (FastPrep; QBiogen, Irvine, CA) for six 30 second pulses, each with a 30 second interval on ice in between pulses. Centrifugation removed bead and cell debris. The soluble fraction was kept at −80°C until further analysis. Bio-Rad protein assay kit (BioRad, Valencia, CA) measured protein concentration.

Reduction used 4 μl of 450 mM dithiothreitol (DTT, Sigma-Aldrich, ST. Louis, MO) added to 25 μl of supernatant and incubated for 45 min at 55°C. Digestion required 2.5 μg of mass spectrometry grade trypsin (Promega, Madison, WI) added to the reduced protein mixture and incubated overnight at 37°C. The tryptic peptides were then purified by C18 Ziptip (Millipore, Billerica, MA) according to the manufacturer’s manual. Briefly, the Ziptip was first prepared by washing with 50% acetonitrile (ACN)/H_2_O followed by 0.1% (v/v) trifluoroacetic acid (TFA) in H_2_O. The tryptic peptide solution was then loaded onto the Ziptip and washed with 0.1% (v/v) TFA in H_2_O. The peptides were eluted with 50% ACN in H_2_O. The purified sample was dried prior to MS analysis.

### Protein identification

Digested samples were analyzed by the Genome Center Proteomics Core at the University of California, Davis. Protein identification was performed using an Eksigent Nano LC 2-D system (Eksigent, Dublin, CA) coupled to a LTQ ion trap mass spectrometer (Thermo-Fisher, San Jose CA) through a Picoview Nano-spray source. Peptides were loaded onto an Agilent nanotrap (Zorbax 300SB-C18, Agilent Technologies) at a loading flow rate of 5.0 μL/min. Peptides were then eluted from the trap and separated by a nano-scale 75 μm x 15 cm New Objectives picofrit column packed in house with Michrom Magic C18 AQ packing material. Peptides were eluted using a 90 minute gradient of 2-80% buffer B (Buffer A = 0.1% formic acid, Buffer B = 95% acetonitrile/0.1% formic acid). The top 10 ions in each survey scan were subjected to automatic low energy collision-induced dissociation (CID).

For the protein identification among the replicates, a scoring system was developed to determine the presence of each protein from datasets where one biological replicate identifies a protein and another does not. Three parameters were evaluated to score the presence of a particular protein: (a) the number of unique peptides (*Pep*_*uniq*_) used for the identification of a protein, (b) the probability values from X! Tandem (−log (e)) and (c) the number of times a protein showed up in all three biological replicates. First, proteins were scored as described in Table 3. Then, if the cumulative score of a protein in the three biological replicates was greater than or equal to three the same number of replicates, it was considered to be present in the sample. For example, if the protein was identified with high confidence (*Pep*_*uniq*_ ≥ 2 and –log (e) ≥ 10) in at least one of the three replicates or with low confidence (*Pep*_*uniq*_ = 1 and 2 ≤ −log (e) ≤ 10) in all three replicates, the score will be three and thus considered to be present in the sample.

**Table 3:** **Protein scoring system for the protein determination in replicates**

^**a**^ **Pep**_**uniq**_		^**b**^ **-Log (e)**	**Score (** ***S*** **)**
≥2	**AND**	≥10	3
≥2	**OR**	≥10	2
=1	**AND**	2 ≤ −Log(e) < 10	1

^a^Pep_uniq_ is the number of unique peptide used to the protein identification.

^b^-Log (e) is the expectation value of protein identification by X!Tandem.

Identification score of protein k (*ID_S*(*P*_*k*_)) was calculated as a sum of each scores obtained from each replicates. When n = 3 (triplicate), protein with *ID_S*(*P*_*k*_) ≥ 3 was considered present in a sample.

### Database searching and false discovery rate (FDR)

Tandem mass spectra were extracted and charge states were deconvoluted by BioWorks version 3.3. All MS/MS samples were analyzed using X! Tandem (www.thegpm.org). X! Tandem was set up to search against the *L. lactis* whole proteome with protein supplements expressed from heterologous plasmids. X! Tandem searched with a fragment ion mass tolerance of 0.60 Da, and specified methionine oxidation as a variable modification. Peptide false discovery rates (FDR) were determined by independent MS/MS spectra searches against forward (target) and reverse (decoy) database of *L. lactis* IL1403 (including plasmid proteins). FDR was calculated as R/(F + R) where R and F were the number of peptides from decoy and target databases. The search was performed at a fixed 1% FDR level.

*Bioinformatics*- Protein functionality coded on *L. lactis* IL1403 were obtained from NCBI (http://www.ncbi.nlm.nih.gov) and JGI (http://img.jgi.doe.gov) [17].

### Calculation of relative number of total spectra (*R*_*TS*_)

Quality assessment of LC-MS/MS datasets between two samples. Relative number of total spectra (*R*_*TS*_) was determined using equation 1, where *SpC*_*A,i*_ corresponds to the number of spectra for the protein *i* in sample A, N_A_ and N_B_ are the number of proteins in sample A and B, respectively.

1 $$ {R}_{TS}=\frac{Max\left({\displaystyle \sum_{i=1}^{N_A}Sp{C}_{A,i},{\displaystyle \sum_{i=1}^{N_{{}_B}}Sp{C}_{B,i}}}\right)}{Min\left({\displaystyle \sum_{i=1}^{N_A}Sp{C}_{A,i},{\displaystyle \sum_{i=1}^{N_{{}_B}}Sp{C}_{B,i}}}\right)} $$

*R*_*TS*_ is the ratio of total number of tandem mass spectra used for the identification of proteins in the sample A and B. It has a value larger than or equal to, 1.0.

### Calculation of relative quantification between independent samples

The relative amount of a specific protein between samples A and B was calculated using the number of tandem mass spectra of the specific protein and the internal standards. Nine glycolytic enzymes involved in carbohydrate catabolism were used as internal standards (Table 4). The NSAF of protein *k* in sample A (*P*_*A,k*_) was divided by the NSAF of internal standard *j* of sample A (*IN*_*A,j*_). To calculate the ratio of protein *k* between sample A and B, the normalized value of protein *k* in sample A was divided by the value of the same protein *k* in sample B. Since we employ nine internal standards, the resulting ratios were averaged. However, ratios could not be directly averaged. For example, a ratio of 2.0 corresponds to a two-fold increase and a ratio of 0.5 corresponds to a two-fold decrease. Thus the net average change of two replicates, which gives a two-fold increase and a two-fold decrease, respectively, should be zero. However, arithmetically, the average ratio of the example above would be 1.25, which is incorrect. To convert the ratio to the linear scalar value the scalar relative amount (SRA) was defined.

**Table 4:** **Internal standards used in this study**

**gi number**	**Symbol**	**Name**
15674150	*pgiA*	glucose-6-phosphate isomerase
15673315	*pfkA*	6-phosphofructokinase
15673891	*pbaA*	fructose-bisphosphate aldolase
15673116	*tpiA*	triosephosphate isomerase
15674228	*gapA*	glyceraldehyde 3-phosphate dehydrogenase
15672227	*pgk*	phosphoglycerate kinase
15672318	*pmg*	phosphoglyceromutase
15672626	*eno*	phosphopyruvate hydratase
15673314	*pyk*	pyruvate kinase

2 $$ SRA\left[\alpha \Big|\beta \right]=\left\{\begin{array}{c}\hfill \alpha \ge \beta,\ \left(\alpha /\beta \right)-1\hfill \\ {}\hfill \alpha <\beta,\ 1-\left(\beta /\alpha \right)\hfill \end{array}\right. $$

Where α and β are the two values or functions in the ratio that we wish to calculate. In this equation, plus and minus only indicate the direction of the change. For the description of relative amount (ratio), a value of one-fold has to be added to the value of *SRA* [α| β]. For instance, *SRA* [α| β] of +0.5 and −0.5 corresponds to a 1.5-fold increase and decrease, respectively.

Using this definition, the SRA of protein *k* between two replicates A and B (*SRA*[*rep, k*]) can be described as follows where *P*_*replicate*__*A,k*_ is the amount of protein *k* in replica A.

3 $$ SRA\ \left[ NSAP\ \left({P}_{replicate\ A,\ k}\right)\Big| NSAF\ \left({P}_{replicate\ B,\ k}\right)\right] = SRA\ \left[SpC\left({P}_{replicate\ A,\ k}\right)\Big|SpC\ \left({P}_{replicate\ B,\ k}\right)\right] $$

In this equation, A and B indicates samples and *k* represents a protein. A and B can be replicates of one sample in a same condition (*SRA*[*rep, k*]) or two samples from different biological conditions (*SRA*[*comp, k*]). When the protein *k* is an internal standard, it is designated as *SRA*[*A|B, IN*_*j*_] and then used to evaluate the comparability between two samples.

### Calculation of GFP expression using internal standards

In order to adjust the biological variability, a set of internal standard has been used to calculate the GFP expression. The adjusted SRA of protein expression between sample A and B (*SRA* [A|B, *k*]_*adj*_) was calculated as follows where *IN*_*A,j*_ is the *j*th internal standard in sample A and N is the total number of internal standards (N = 9 in work).

4 $$ SRA\ {\left[\mathrm{A}\Big|\mathrm{B},\ k\right]}_{adj} = \frac{1}{N}{\displaystyle \sum_{j=1}^N}SRA\ \left[\frac{NSAF\left({P}_{A,\ k}\right)}{NSAF\ \left(I{N}_{A,\ j}\right)}\ \Big|\frac{NSAF\left({P}_{B,\ k}\right)}{NSAF\ \left(I{N}_{B,\ j}\right)}\ \right] $$

Because the same internal standard *j* (*IN*_*j*_) and protein *k* (*P*_*k*_) are used to calculate the SRA, equation (3) can be simplified as follows and depends solely on the number of spectra.

5 $$ SRA\ {\left[\mathrm{A}\Big|\mathrm{B},\ k\right]}_{adj} = \frac{1}{N}{\displaystyle \sum_{j=1}^N}SRA\ \left[\frac{SpC\left({P}_{A,\ k}\right)}{SpC\ \left(I{N}_{A,\ j}\right)}\ \Big|\frac{SpC\ \left({P}_{B,\ k}\right)}{SpC\ \left(I{N}_{B,\ j}\right)}\ \right] $$

### Statistical analysis

Student *t*-test was used to compare GFP expression’s relative increase calculated using LC-MS/MS and external measurements using fluorescence. The *t*-test was performed with two-tailed and two samples with unequal variance (heteroscedastic) conditions.

### Availability of supporting data

The mass spectrometric datasets used in this experiments and the corresponding GPM protein identification results were available on the ProteomeXchange site (www.proteomexchange.org) with the submission reference of 1-20150322-14021.

## Additional files

Additional file 1: Table S1. The relative amount of total protein and the internal standard between replicates. **Table S2.** Protein concentrations of crude cell lyzates grown in different carbon source. **Table S3.** The average relative amounts of internal standards between wild type and KE mutant strains of *Oryza sativa subsp. japonica*. Experiments were performed in triplicate. **Table S4.** The standard deviation of the relative amounts of internal standards between wild type and KE mutant strain of *Oryza sativa subsp. japonica*s.
Additional file 2: Figure S1. Histogram of the scalar relative amount between replicates (*SRA*[*rep, k*]). Each graph contains the accumulated result s of the total proteins in the comparison between replicates. Zero indicates the same quantity (1.0 fold) and red line represents a simulation of the Normal/Gaussian distribution. **Figure S2.** The correlation of the average relative amount of internal standards between replicates and *R*_*TS*_. No linear correlation was observed (*r* ^*2*^ = 0.486). **Figure S3.** The log-log plate of NSAF of total proteins between replicates. The comparison between replicates A/B, B/C and A/C are represented as circle, reverse triangle and square, respectively. The linear regression coefficient of each comparison is listed next to the symbol in parenthesis. **Figure S4.** Linear correlation between *SD_SRA*[*rep, IN*_*i*_] and the *R*_*TS*_ values. Mass spectrometric data were retrieved from the ProteomeXchange website. *Escherichia coli* whole cell proteome of six replicates were compared for their reproducibility. The expression of protein were determined by the GPM machine with the FDR less than 0.75%. Protein identification within six replicates were determined by the algorithm described in the Materials and Methods. Since the number of replicates were six in this dataset, the score (*ID_S*(*P*_*K*_)) ≧ 6 were considered present in the sample.

## Abbreviations

*SpC*_*k*_: A number of MS/MS spectra used for the identification of protein *k*
*L*_*k*_: A number of amino acids of the protein *k*
*P*_*A,k*_: The protein *k* in sample A
*IN*_*A,j*_: The *j*th internal standard in sample A
*NSAF*(*P*_*A,k*_): The Normalized spectra abundance factor of protein *k* in sample A
*R*_*TS*_: The relative number of total spectra
*SRA*[*rep, k*]: The scalar relative amount of *P*_*k*_ in replicates
*SRA*[*comp, k*]: The scalar relative amount of *P*_*k*_ in two compared samples
*SRA*[*rep, IN*_*j*_]: The scalar relative amount of the *j* ^*th*^ internal standard (*IN*_*j*_) in replicates
*SRA*[*comp, IN*_*j*_]: The scalar relative amount of the *j* ^*th*^ internal standard (*IN*_*j*_) in two samples compared
*Ave_ SRA*[*rep, k*]: The average of the *SRA*[*rep, k*] of total proteins in replicates
*Ave_ SRA*[*rep, IN*_*j*_]: The average of the *SRA*[*rep, IN*_*j*_] of internal standards in replicates
*SD_ SRA*[*rep, k*]: The standard deviation of the *Ave_ SRA*[*rep, k*]
*SD_ SRA*[*rep, IN*_*j*_]: The standard deviation of the *Ave_ SRA*[*rep, IN*_*j*_]
*Pep*_*uniq*_: The number of unique peptides detected in LC-MS/MS
FDR: A false discovery rate
