# Supplementary material for: Comparative proteomics: assessment of biological variability and dataset comparability
Source: BMC Bioinformatics. 2015 Apr 17;16:121. doi: 10.1186/s12859-015-0561-9 (PMC4704264; doi:10.1186/s12859-015-0561-9)
Supplement: Additional file 1: Table S1. — The relative amount of total protein and the internal standard between replicates. Table S2. Protein concentrations of crude cell lyzates grown in different carbon source. Table S3. The average relative amounts of internal standards between wild type and KE mutant strains of Oryza sativa subsp. japonica. Experiments were performed in triplicate. Table S4. The standard deviation of the relative amounts of internal standards between wild type and KE mutant strain of Oryza sativa subsp. japonicas. [file 12859_2015_561_MOESM1_ESM.docx]

Supplementary Table S1

The relative amount of total protein and the internal standard between replicates

^a^ A/B is the comparison between replicate A and B. The A, B, and C stand for the replicates of each sample.

^b^ TP is the average relative amount of total proteins between replicates which is calculated as (RQ_0_+*Ave_SRA*[*rep, k*]) ± *SD_SRA*[*rep, k*]. RQ_0_ is +1 or -1 if *Ave_SRA*[*rep,k*]<0.

^c^ IS is the average relative amount of internal standard between replicates.

Supplementary Table S2

Protein concentrations of crude cell lyzates grown in different carbon source.

^a^ *Bifidobacterium longum* subsp. *infantis* ATCC15697 were anaerobically cultivated in M17 media with different carbon source (2% (w/v)). Initial cell OD was normalized at 1.0 then 10ml of cells were concentrated. The concentrated cells were resuspended in 1ml of 8M urea/100mM tris buffer and disrupted by bead beater with 300ug of glass bead. Protein concentration was measured by Protein assay kit (Bio-Rad). MS analyses were performed in the same condition described in the text.

^b^ Cells were taken at the early exponential phase.

^c^ HMO: human milk oligosaccharides. Cells were taken at the early stationary phase

^d^ FOS: frutoligosaccharide. Cells were taken at the late stationary phase.

Supplementary Table S3

The average relative amounts of internal standards between wild type and KE mutant strains of Oryza sativa subsp. japonica. Experiments were performed in triplicate.

^a^ All of enzymes involved in the Calvin cycle were found by the LC-MS/MS analysis. The enzymes, whose *SpC_k_* were bigger than 4 were selected as internal standards.

^b, c^ Wild type and KE mutant strain of Oryza sativa subsp. japonica, respectively.

^d^ The relative amounts of obtained as described in the Materials and Methods. The NSAF of two triplicates were compared individually and the relative amounts of individual comparisons were averaged.

Supplementary Table S4

The standard deviation of the relative amounts of internal standards between wild type and KE mutant strain of Oryza sativa subsp. japonicas.

^a^ The individual triplicates of wild type and KE mutant cell were compared. The number affixed indicates the number of each triplicate.

^b^ The total number of spectra were in the range of 1200~1360.

^c^ The *SD_SRA*[*comp, INj*] and the *Ave_SRA*[*comp, INj*] were calculated based on the NSAF of internal standards.
