# Supplementary material for: Comparative proteomics: assessment of biological variability and dataset comparability
Source: BMC Bioinformatics. 2015 Apr 17;16:121. doi: 10.1186/s12859-015-0561-9 (PMC4704264; doi:10.1186/s12859-015-0561-9)
Supplement: Additional file 2: Figure S1. — Histogram of the scalar relative amount between replicates (SRA[rep, k]). Each graph contains the accumulated result s of the total proteins in the comparison between replicates. Zero indicates the same quantity (1.0 fold) and red line represents a simulation of the Normal/Gaussian distribution. Figure S2. The correlation of the average relative amount of internal standards between replicates and R TS. No linear correlation was observed (r 2 = 0.486). Figure S3. The log-log plate of NSAF of total proteins between replicates. The comparison between replicates A/B, B/C and A/C are represented as circle, reverse triangle and square, respectively. The linear regression coefficient of each comparison is listed next to the symbol in parenthesis. Figure S4. Linear correlation between SD_SRA[rep, IN i] and the R TS values. Mass spectrometric data were retrieved from the ProteomeXchange website. Escherichia coli whole cell proteome of six replicates were compared for their reproducibility. The expression of protein were determined by the GPM machine with the FDR less than 0.75%. Protein identification within six replicates were determined by the algorithm described in the Materials and Methods. Since the number of replicates were six in this dataset, the score (ID_S(P K)) ≧ 6 were considered present in the sample. [file 12859_2015_561_MOESM2_ESM.pptx]

## Slide 1
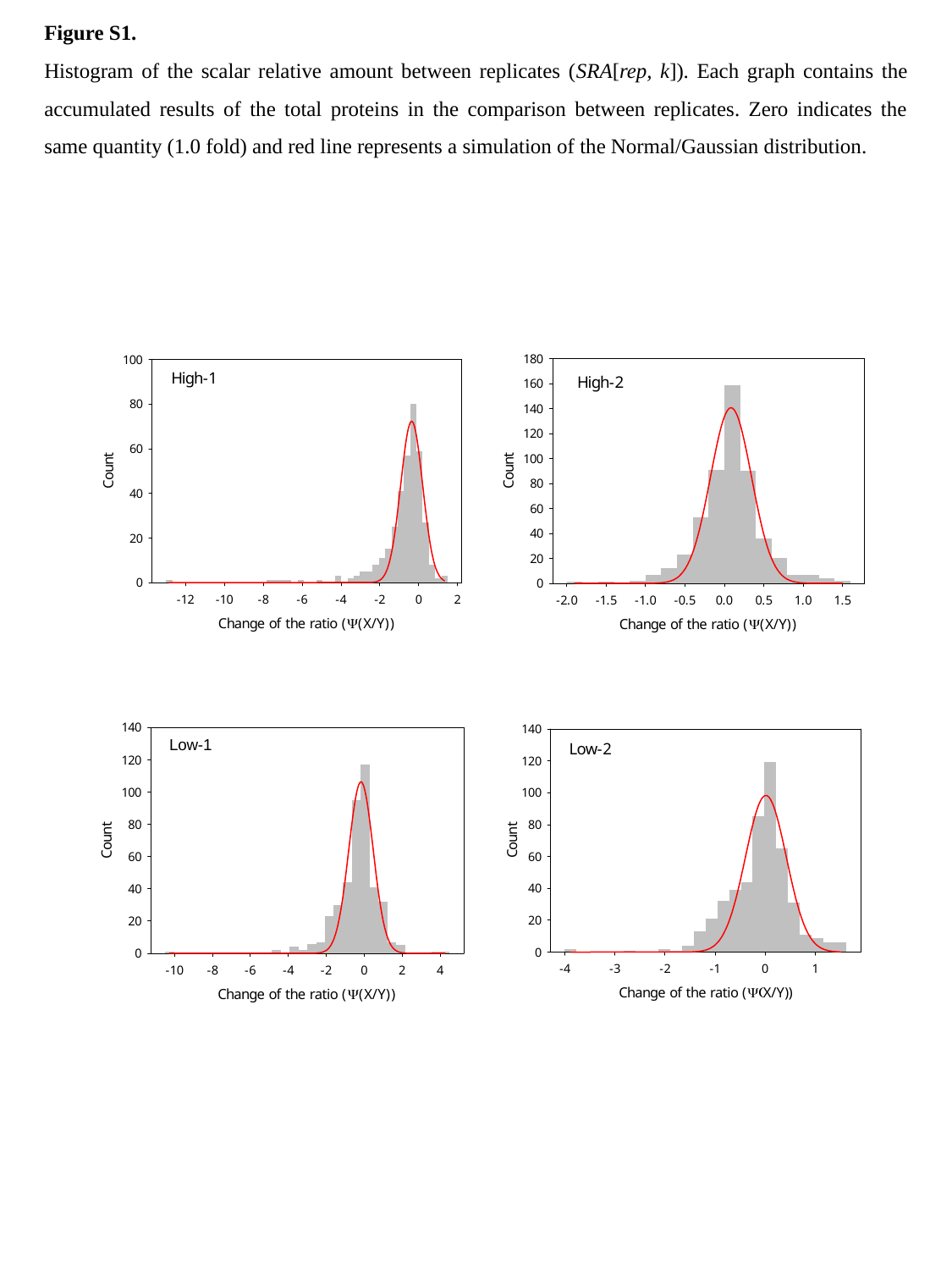

Figure S1.
Histogram of the scalar relative amount between replicates (SRA[rep, k]). Each graph contains the accumulated results of the total proteins in the comparison between replicates. Zero indicates the same quantity (1.0 fold) and red line represents a simulation of the Normal/Gaussian distribution.

## Slide 2
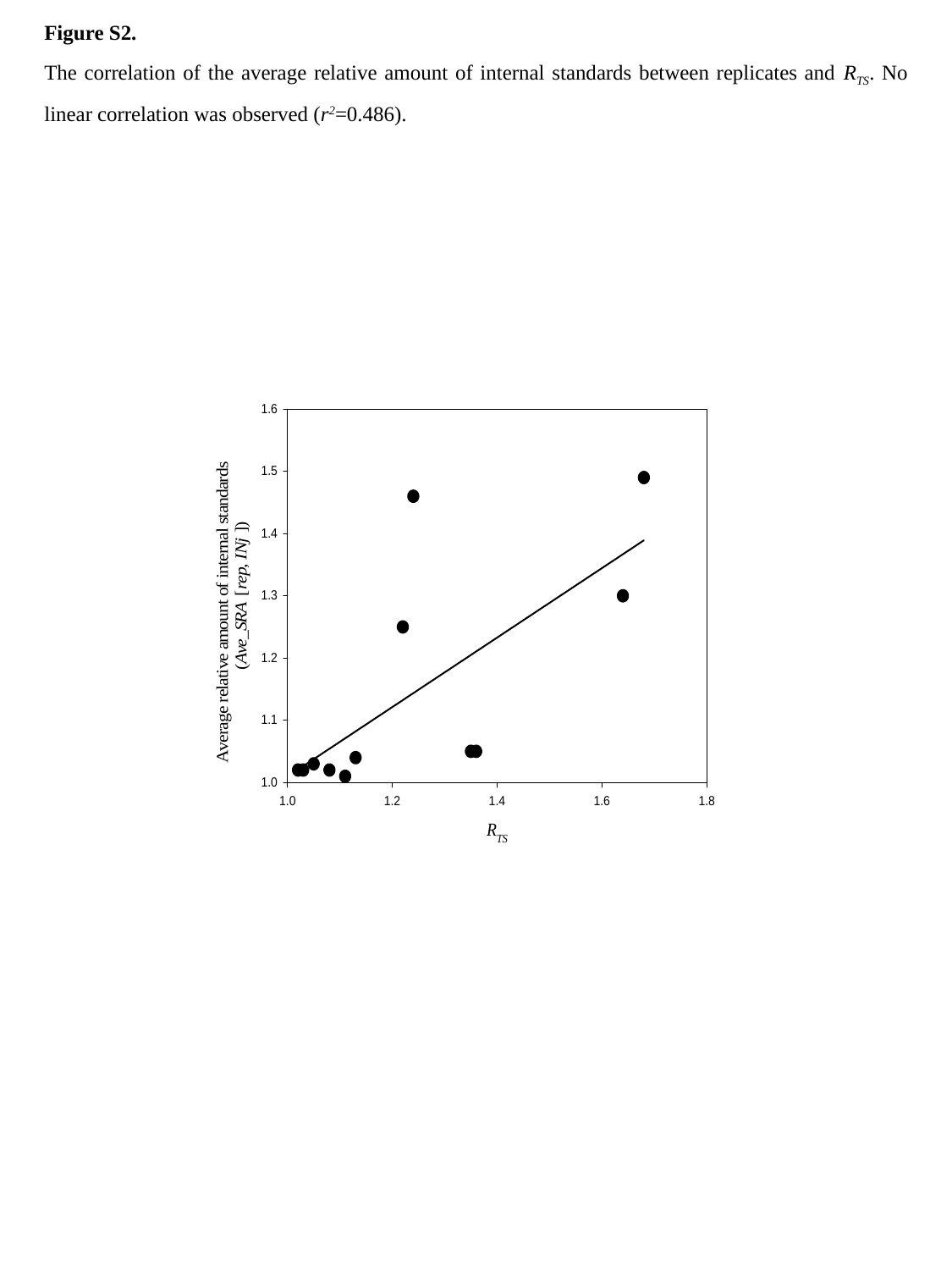

Figure S2.
The correlation of the average relative amount of internal standards between replicates and RTS. No linear correlation was observed (r2=0.486).

## Slide 3
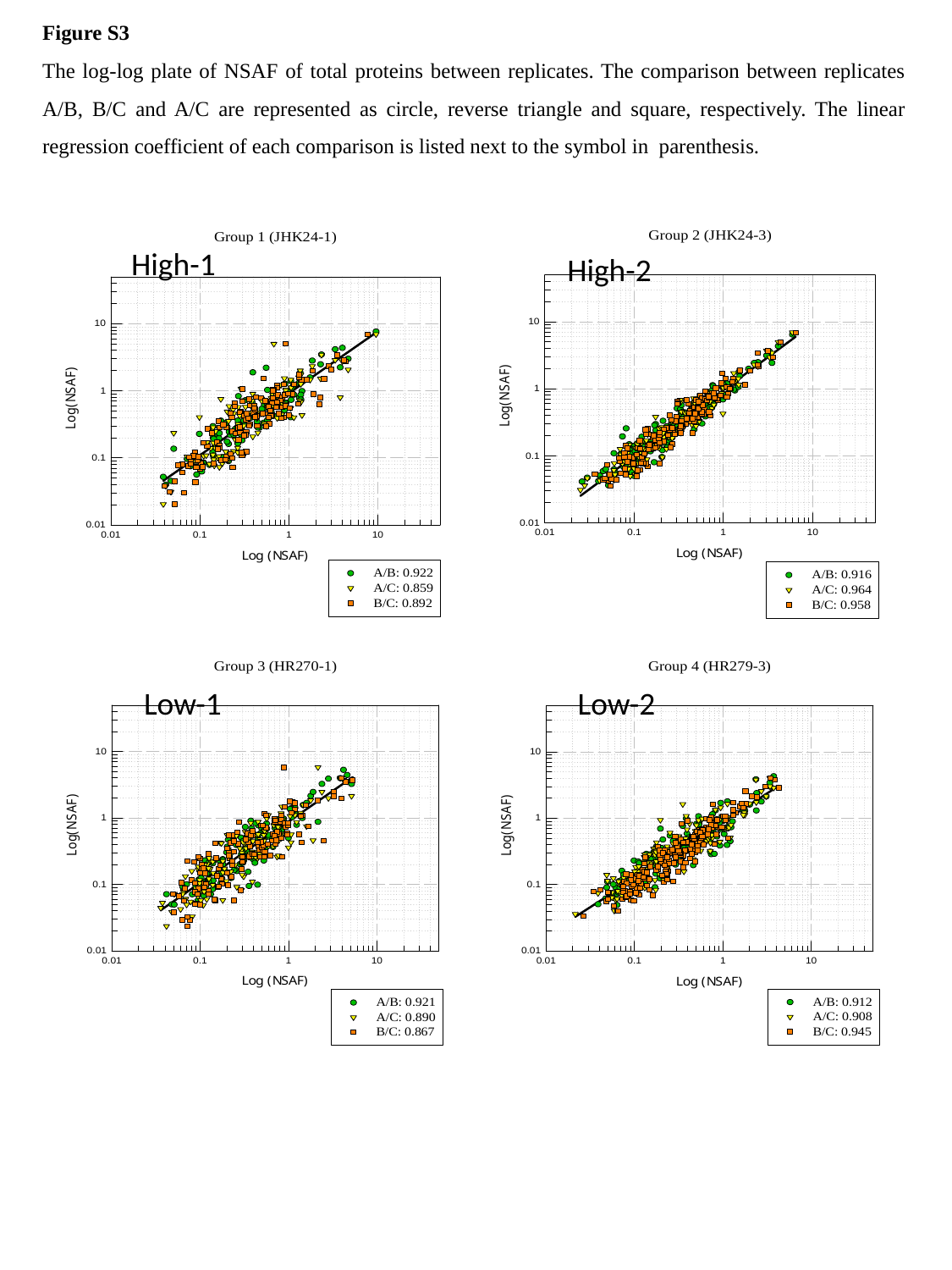

Figure S3
The log-log plate of NSAF of total proteins between replicates. The comparison between replicates A/B, B/C and A/C are represented as circle, reverse triangle and square, respectively. The linear regression coefficient of each comparison is listed next to the symbol in parenthesis.
High-1
High-2
Low-1
Low-2

## Slide 4
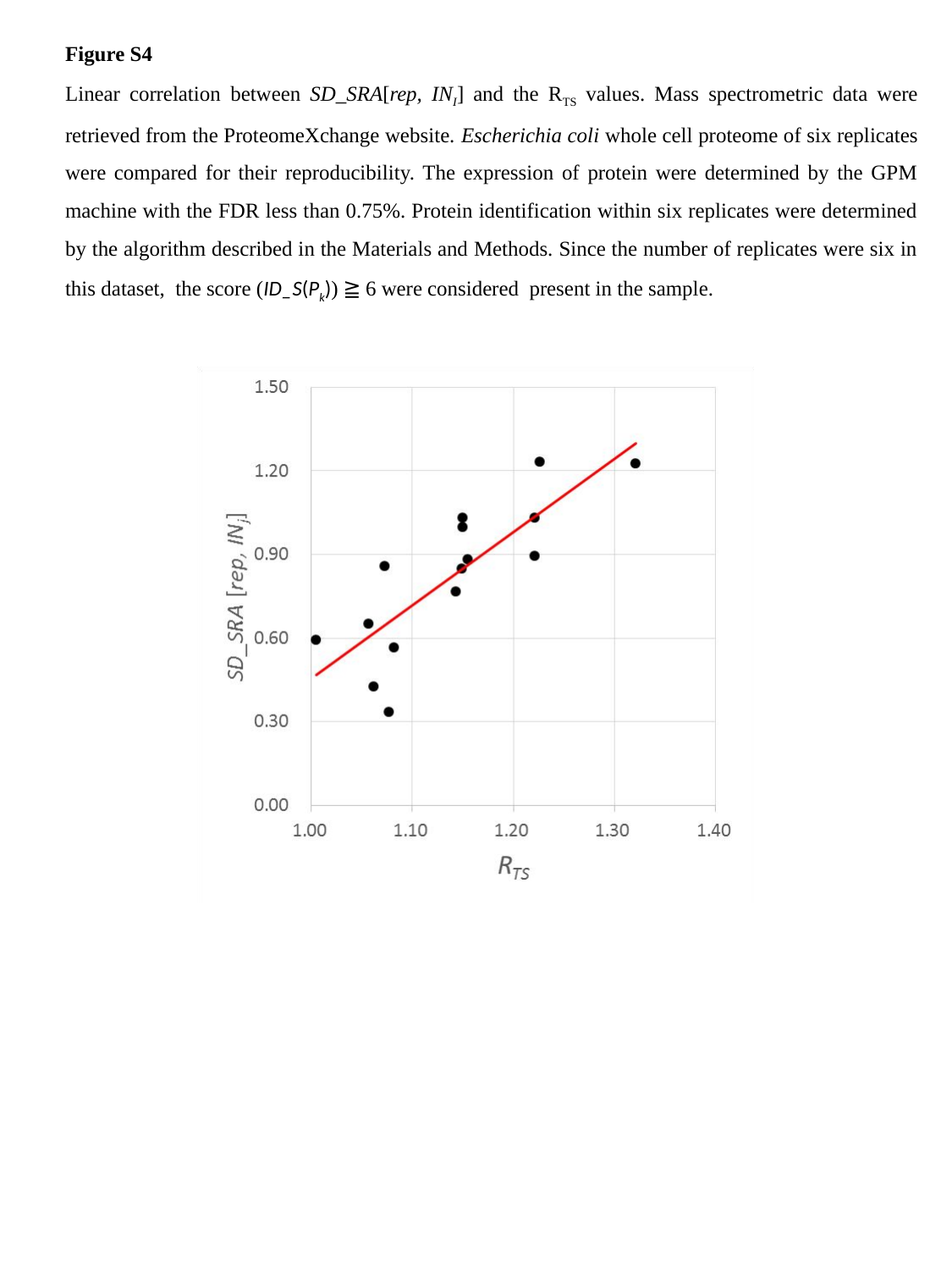

Figure S4
Linear correlation between SD_SRA[rep, INI] and the RTS values. Mass spectrometric data were retrieved from the ProteomeXchange website. Escherichia coli whole cell proteome of six replicates were compared for their reproducibility. The expression of protein were determined by the GPM machine with the FDR less than 0.75%. Protein identification within six replicates were determined by the algorithm described in the Materials and Methods. Since the number of replicates were six in this dataset, the score (ID_S(Pk)) ≧ 6 were considered present in the sample.
